# EXTREME WEATHER AND HEALTH AMONG OLDER POPULATIONS: INDIVIDUAL AND CONTEXTUAL-LEVEL VULNERABILITY AND RESILIENCE

**DOI:** 10.1093/geroni/igad104.3730

**Published:** 2023-12-21

**Authors:** Eunyoung Choi, Bei Wu, Jennifer Ailshire

**Affiliations:** University of Southern California, Los Angeles, California, United States; New York University, New York City, New York, United States; University of Southern California, Los Angeles, California, United States

## Abstract

Intense, frequent extreme weather events pose a significant public health threat, particularly for middle-aged and older adults who may be at elevated health risk. Yet, the social and environmental factors that determine their vulnerability and resilience remain largely unexplored. This study investigates individual and contextual determinants associated with exposure to and the health effects of extreme weather. Data were from the 2021 California Health Interview Survey of adults aged 50+ (N=14,726). Respondents were asked whether they had experienced any hazardous weather events such as heat waves, floods, and wildfires in the past two years and whether mental or physical health was harmed by these events. About 53% were exposed to extreme weather events. Among the exposed group, 18% and 17% reported harm to mental and physical health, respectively. Logistic regression analysis revealed increased exposure risk among middle-aged adults (age 50-64), those with chronic conditions, people living alone, and residents of rural and unsafe neighborhoods. Similarly, mental and physical health harms were more prevalent among these groups, along with females and those with unstable housing conditions. Conversely, living in cohesive neighborhoods was identified as a resilient factor, associated with reduced mental (OR=0.78, p< 0.05) and physical health risks (OR=0.70, p< 0.01). This study reveals pronounced vulnerabilities to climate change risks among populations identified as socioeconomically disadvantaged. Moreover, the findings emphasize the role of contextual conditions in shaping vulnerability and resilience. Our study contributes valuable insights to develop targeted climate change mitigation strategies, fostering resilience among older populations and supportive communities.

